# Supplementary material for: T cell receptor β-chains display abnormal shortening and repertoire sharing in type 1 diabetes
Source: Nat Commun. 2017 Nov 27;8:1792. doi: 10.1038/s41467-017-01925-2 (PMC5702608; doi:10.1038/s41467-017-01925-2)
Supplement: Supplementary file 1 — Supplementary Information [file 41467_2017_1925_MOESM1_ESM.pdf]

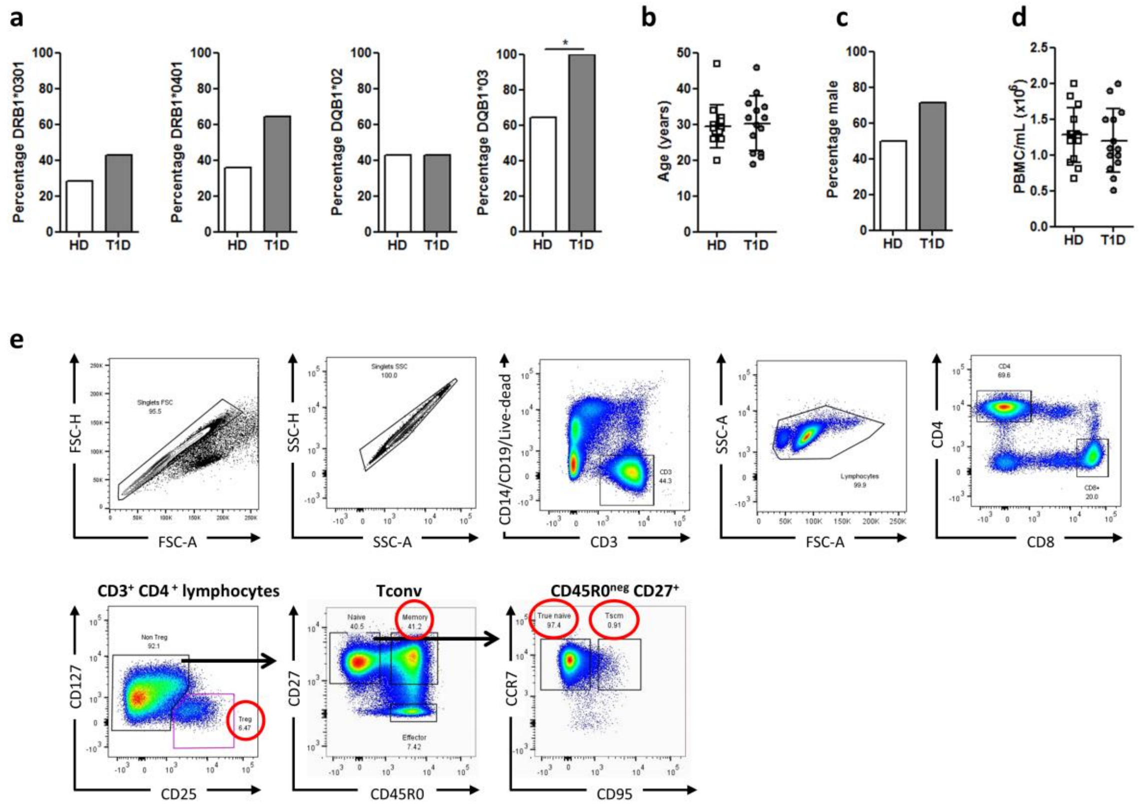

Supplementary Figure 1. Characteristics of the study cohorts and flow cytometry sorting strategy. a-d. There are no differences in the frequency of type 1 diabetes-predisposing HLA alleles (with the only exception of DQB1*03) (**a**), age (**b**), gender (**c**) or PBMC yield from blood (**d**) between healthy donors (HD) and type 1 diabetes patients (T1D). **(e)** Sorting strategy: PBMCs were isolated and stained as described in the Methods section. FSC and SSC doublets and all singlets falling in the dump channel (live/dead, CD14, CD19) were excluded. Live $CD3^+$ cells were gated using FSC and SSC, and then $CD4^+CD8^-$ cells selected. The $CD3^+$ $CD4^+$ $CD25^{hi}$ $CD127^{lo}$ cells were sorted as regulatory T cells (Treg). The remaining conventional T cells were gated using CD45R0 and CD27, and the central memory (CM) cells sorted ($CD45R0^+$ $CD27^+$). Naïve cells ($CD45R0^-$ $CD27^+$) were gated into CCR7 and CD95, and the true naïve (TN) ($CCR7^+$ $CD95^-$) and stem-cell like memory (Tscm) ($CCR7^+$ $CD95^+$) cells sorted (see red circles). **a,c:** Fisher's exact test. **b:** Mann-Whitney U test. Lines represent the mean ± SD. **d:** unpaired t-test. Lines represent the mean ± SD.

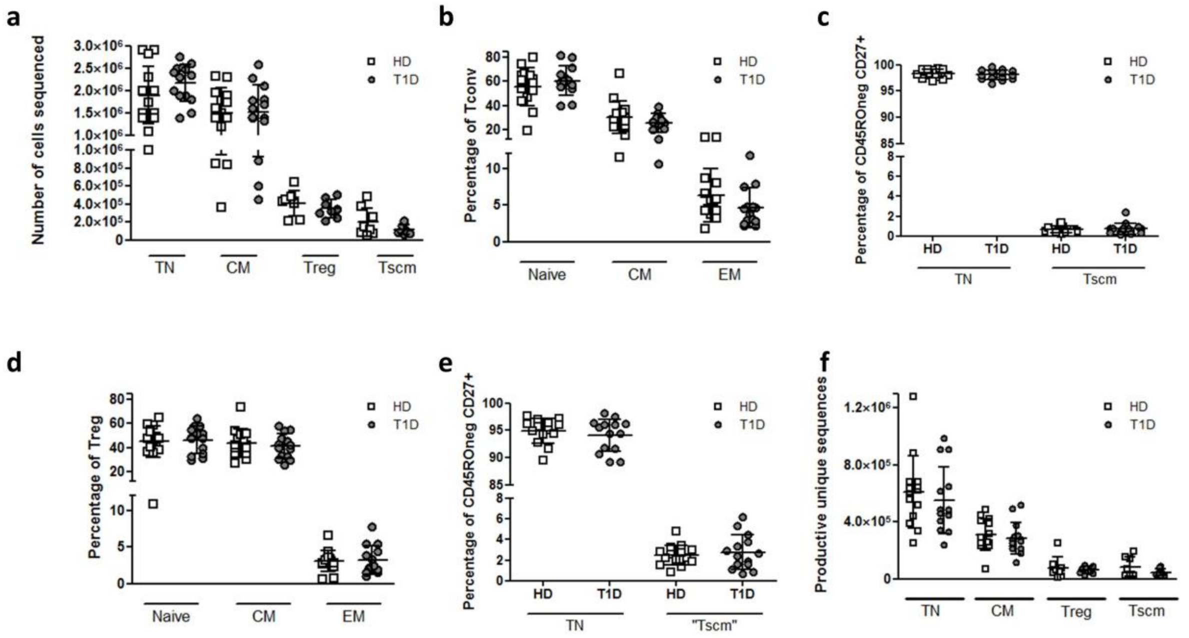

Supplementary Figure 2. Cell and sequencing metrics are comparable between type 1 diabetes patients and healthy donors. a. There were no differences between type 1 diabetes patients (T1D) and healthy donors (HD) in the number of cells sorted and sequenced for each cell subset. **b-e.** There were no differences in the phenotype of the sorted T conventional (Tconv) (**b,c**) and Treg (**d,e**) cells between controls and patients. **b,d** show the percentage of cells in each of the phenotypes assigned by the expression of CD45R0 and CD27 (naïve, CM, effector memory-EM-). **c,e** show the percentage of $CD45R0^-$ $CD27^+$ cells falling in the $CCR7^+$ $CD95^-$ gate (TN) or in the $CCR7^+$ $CD95^+$ gate (Tscm). **f.** There were no differences in the number of productive unique sequences (unique clonotypes) between healthy donors and type 1 diabetes patients for any of the 4 cell subsets. **a-f:** Mann Whitney U test or unpaired t-test. Lines represent the mean ± SD.

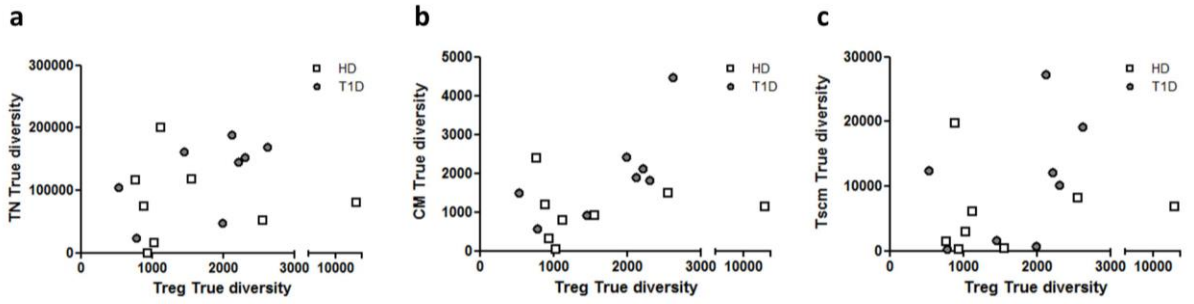

Supplementary Figure 3. Correlations of the diversity indices of Treg cells. The correlations of the true diversity indices were calculated for the Treg cells *versus* true naïve (TN) (**a**), central memory (CM) (**b**) and stem-cell like memory (Tscm) (**c**) cells. None of the correlations is significant (Pearson correlation).

a

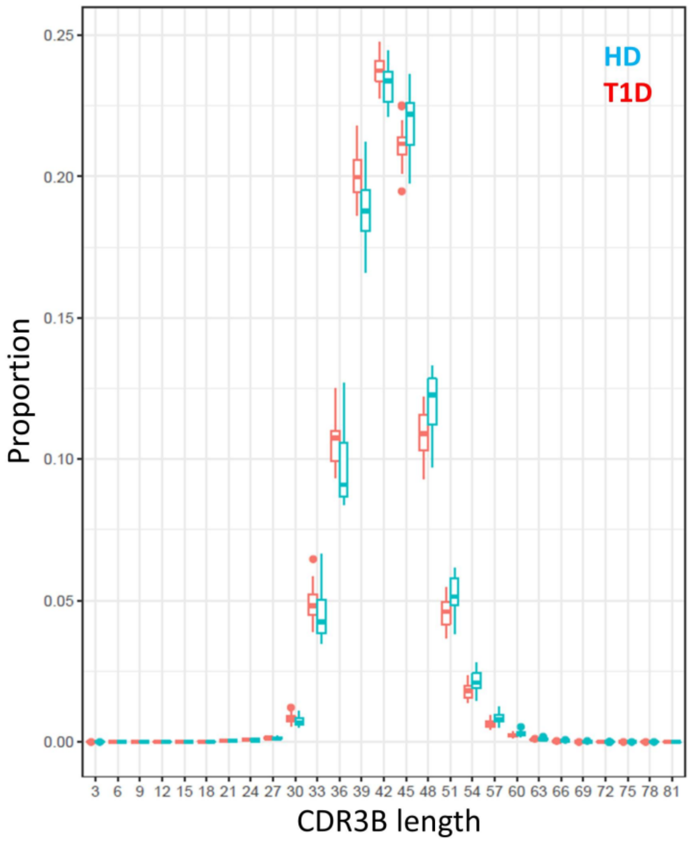

b

Length	p-value
27	0.45
30	0.16
33	0.11
**36**	**0.03**
**39**	**0.01**
42	0.11
**45**	**0.04**
**48**	**0.01**
**51**	**0.02**
**54**	**0.03**
**57**	**0.02**
**60**	**0.03**

Supplementary Figure 4. Randomized subsampling analysis of TCRB CDR3 length distributions. To test whether observed TCRB CDR3 length differences are not an artefact of sub-sampling of true naïve (TN) cells and variable number of reads among samples with resampling analysis, we performed binomial sampling of the number of sequences in the least abundant sample for each sample, for TN cells. This was repeated 500 times and TCRB CDR3 length distributions were computed for each replication. (**a**) TCRB CDR3 length distributions show a shift towards shorter clonotypes in type 1 diabetes patients (T1D) *versus* healthy donors (HD). (**b**) Frequencies of occurrence of each length were compared between type 1 diabetes patients and healthy donors with Mann-Whitney U test.

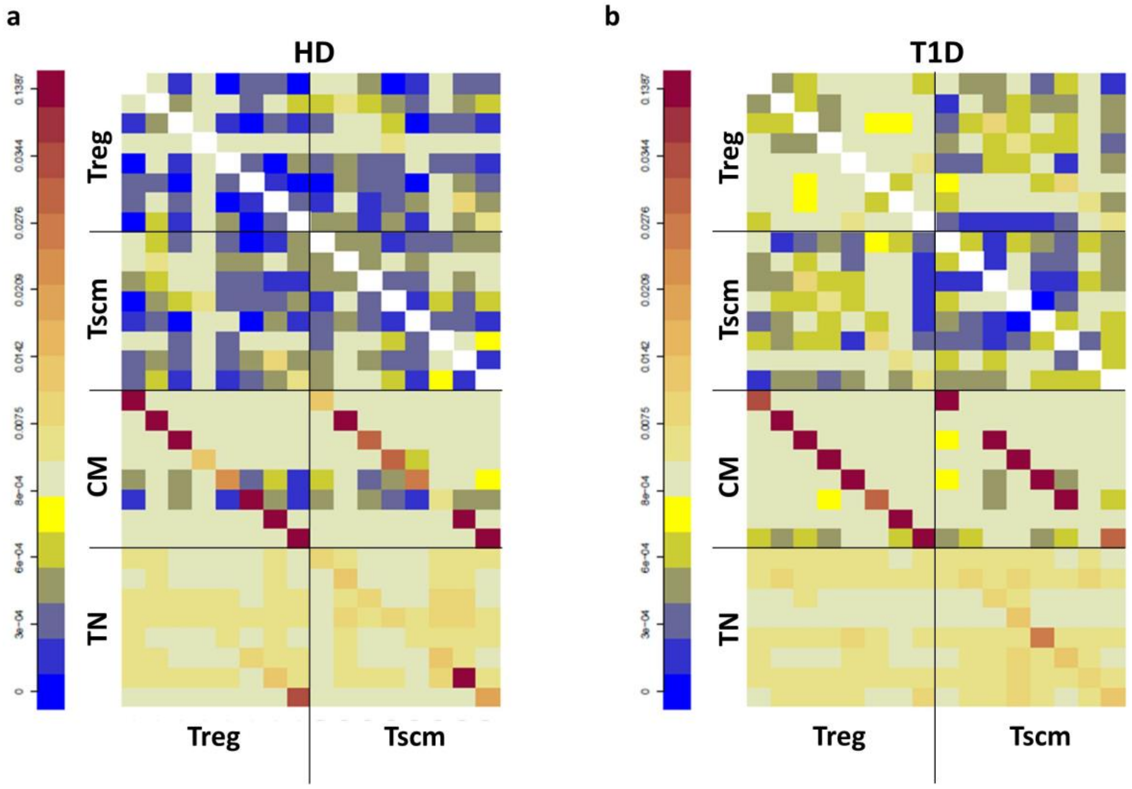

Supplementary Figure 5. Comparison of Treg and Tscm overlap with all other cell subsets. Heatmaps showing the overlap indices for Treg and Tscm TCRB nucleotide repertoires *versus* those of all other cell subsets from 8 healthy donors (**a**) and 8 type 1 diabetes patients (**b**) as described in the Methods section.

.

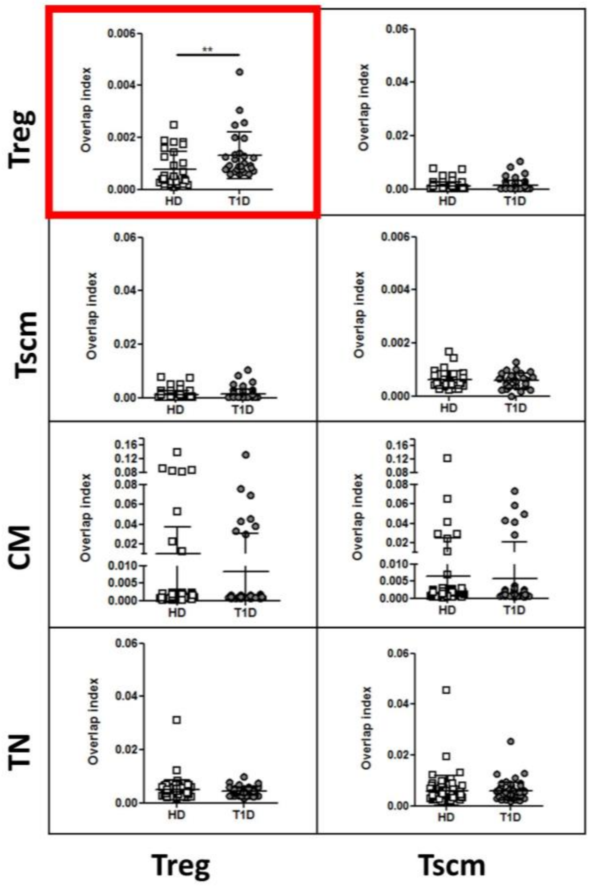

Supplementary Figure 6. Comparison of Treg and Tscm overlap with all other cell subsets. Overlap indices from Supplementary Figure 7. Red border lines denote comparisons where overlap indices are significantly higher in patients. Mann-Whitney U test. **p<0.01. Lines represent the mean ± SD.

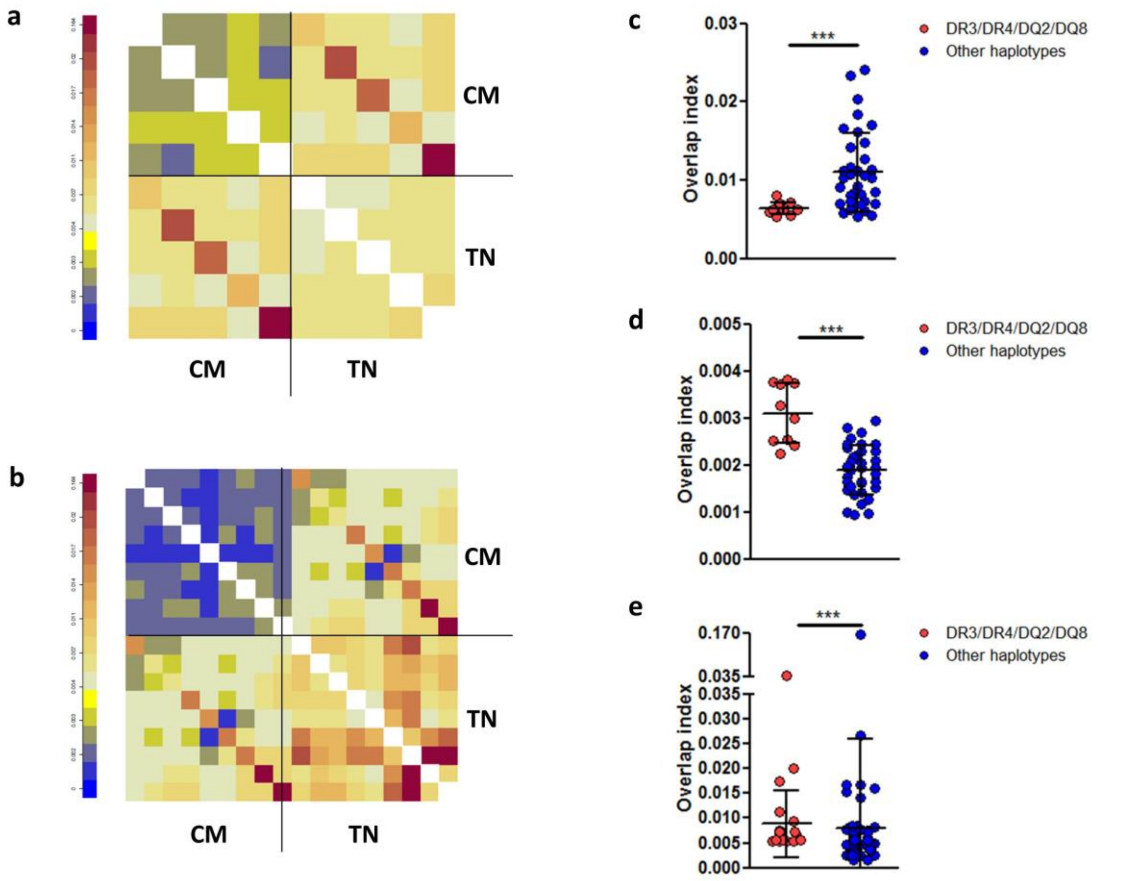

Supplementary Figure 7. Analysis of influence of HLA haplotypes on overlap indices. a-b: Heatmaps plotting the overlap indices at the CDR3 nucleotide level for the five patients with the type 1 diabetes-predisposing haplotype *DRB1*03/DRB1*0401/DQB1*02/DQB1*03* (DR3/DR4/DQ2/DQ8) (**a**) *versus* the 9 patients with other haplotypes (**b**). **c-e.** Overlap indices among TN/TN (**c**), CM/CM (**d**) and TN/CM (**e**) cell subtypes. Mann-Whitney U test. ***p<0.001. Lines represent the mean ± SD.

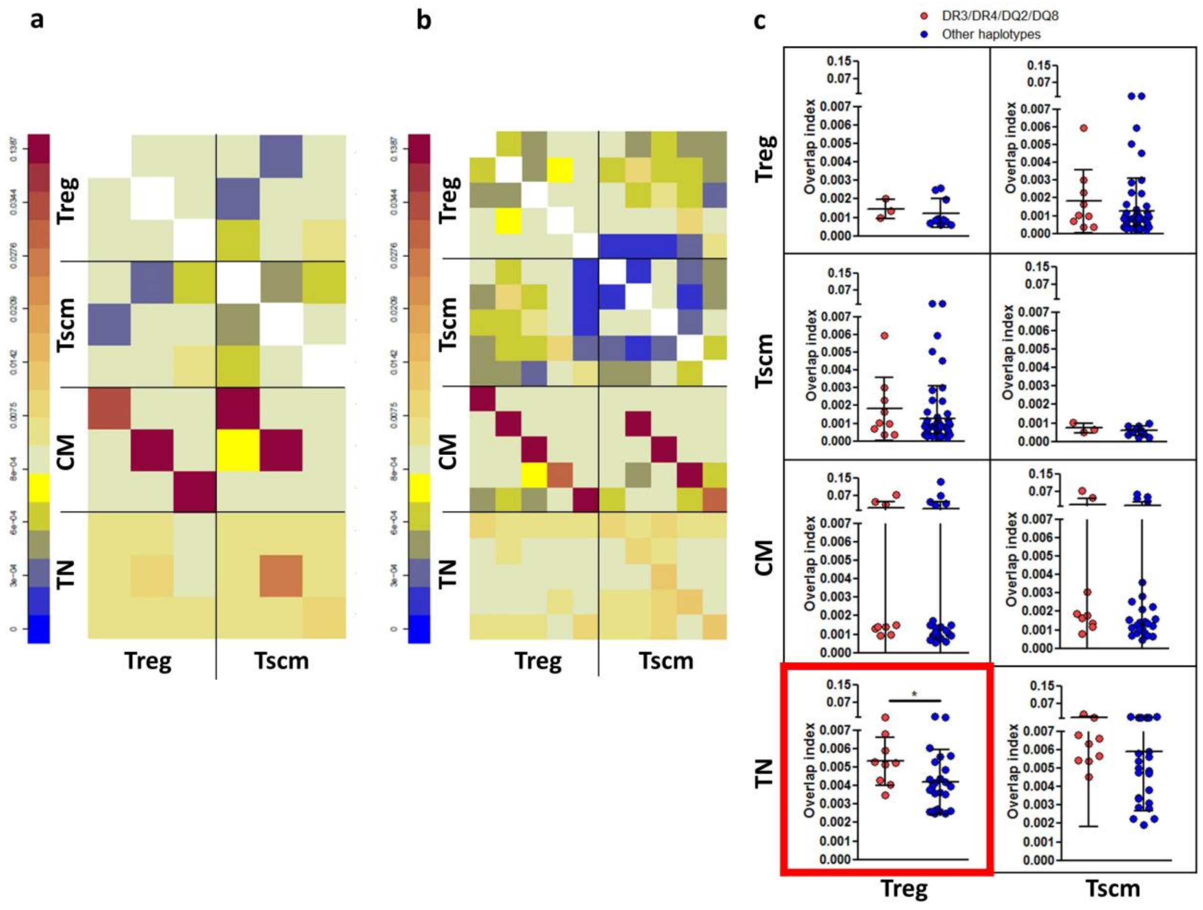

Supplementary Figure 8. Analysis of influence of HLA haplotypes on Treg and Tscm overlap indices. (**a-b**) Overlap indices were calculated for Treg and Tscm TCRB nucleotide repertoires versus those of all other cell subsets for the five patients with the type 1 diabetes-predisposing haplotype *DRB1*03/DRB1*0401/DQB1*02/DQB1*03* (DR3/DR4/DQ2/DQ8) (**a**) *versus* the 9 patients with other haplotypes (**b**). **c.** Overlap indices for all possible comparisons between Treg and Tscm TCRB nucleotide repertoires and all other cell types. Red border lines denote comparisons where overlap indices are significantly higher than those of patients with other haplotypes. Mann-Whitney U test. *p<0.05. Lines represent the mean ± SD.

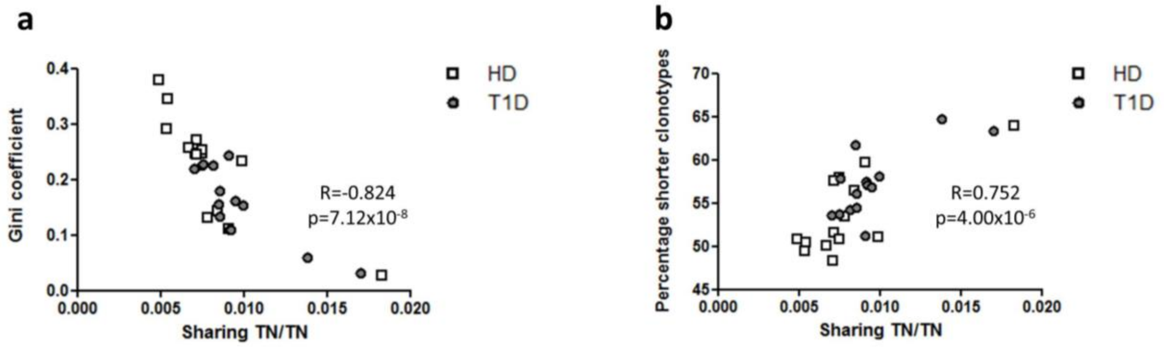

Supplementary Figure 9. Sharing indices correlate with clonality and frequencies of shorter clonotypes. The mean sharing indices of each individual's true naïve (TN) TCR repertoire with all other subjects in their cohort (healthy donors or type 1 diabetes patients) ("sharing TN/TN") correlate with Gini coefficient in TN cells (**a**) and percentage of shorter clonotypes in TN cells (**b**). Pearson correlation. Percentage of shorter clonotypes refers to the percentage of clonotypes between 27 and 42 nucleotides (see Methods).

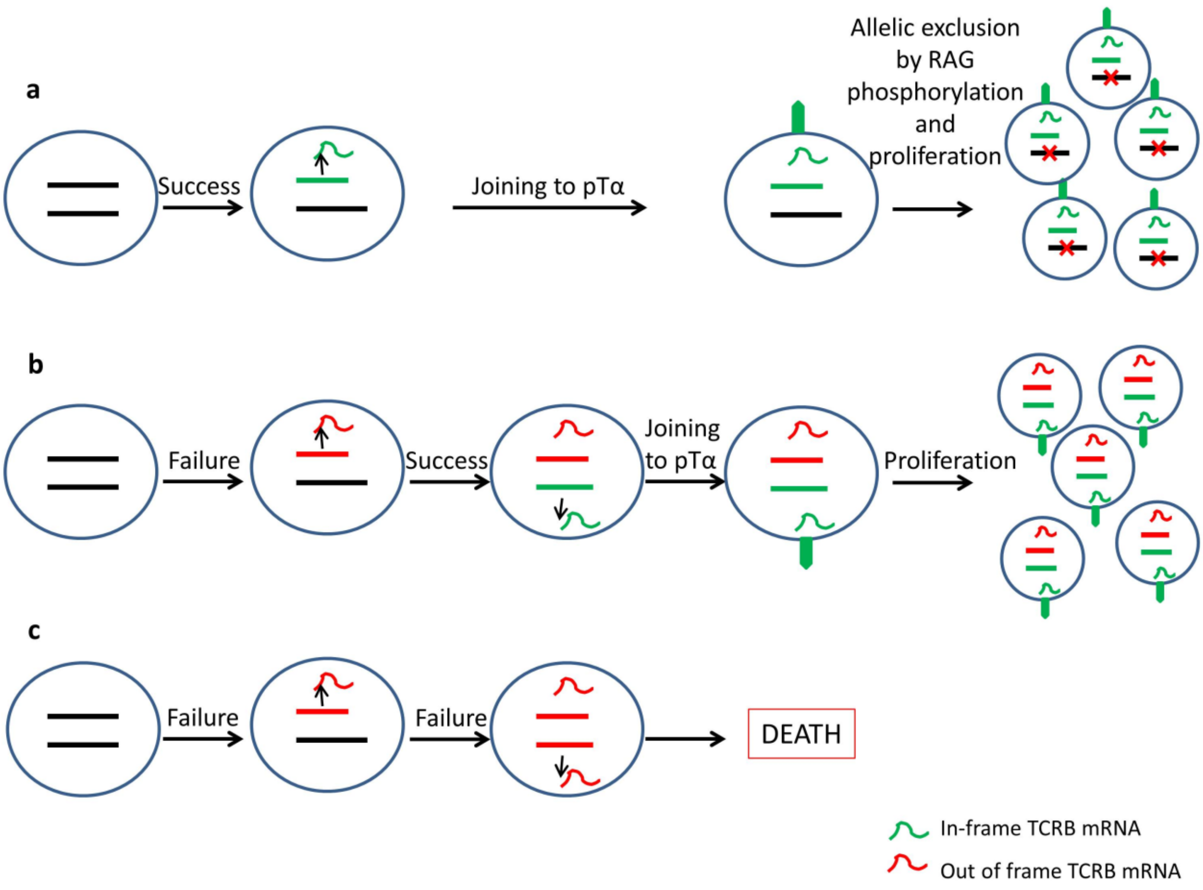

Supplementary Figure 10. Thymic TCRB rearrangement follows one of 3 possible courses. a. TCRB *VDJ* genes successfully rearrange in one of the two chromosomes, leading to a mRNA (green curved line) that translates into a productive TCRB chain, which binds to the surrogate pre-Tα chain, giving rise to the expression of a pre-TCR in the cell surface; this induces a signal that leads to allelic exclusion in the other chromosome via recombination-activating gene (RAG) phosphorylation. Afterwards cells with a functional pre-TCR proliferate, and once the proliferation stops the TCRA rearrangement takes place, followed by positive and negative selection. **b.** If the rearrangement on the first chromosome leads to an out of frame (OOF) mRNA (red curved line), rearrangement proceeds on the second chromosome. If this second rearrangement is successful the cell will express a pre-TCR, and will undergo proliferation, TCRA rearrangement, and positive and negative selection as described above. This cell could then express 2 different TCR mRNAs, one of them OOF. **c.** If rearrangement in both chromosomes leads to OOF mRNAs the cell will not signal through the pre-TCR and will die.

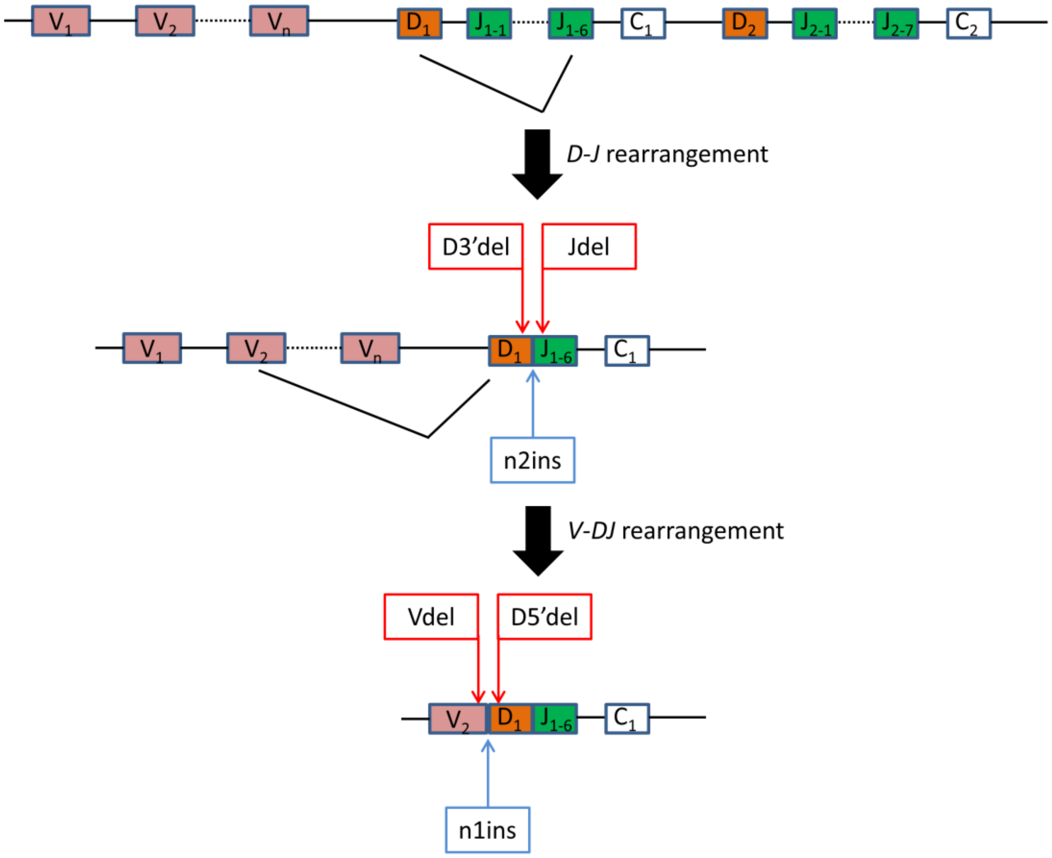

Supplementary Figure 11. TCRB rearrangement process. In a first stage a *D* gene rearranges with a *J* gene; in this process nucleotides are randomly deleted from the 3' end of the *D* gene (D3dels) and from the 5' end of the *J* gene (Jdels). Then the non-homologous end-joining (NHEJ) pathway repairs the break and inserts a random number of nucleotides to the *D-J* junction (n2ins). Once a *DJ* segment is formed it rearranges with a *V* gene, where nucleotides are randomly deleted from the 5' end of the *D* gene (D5dels) and from the 3' end of the *V* gene (Vdels). Finally, the NHEJ pathway adds a random number of nucleotides to the V-DJ junction (n1ins).

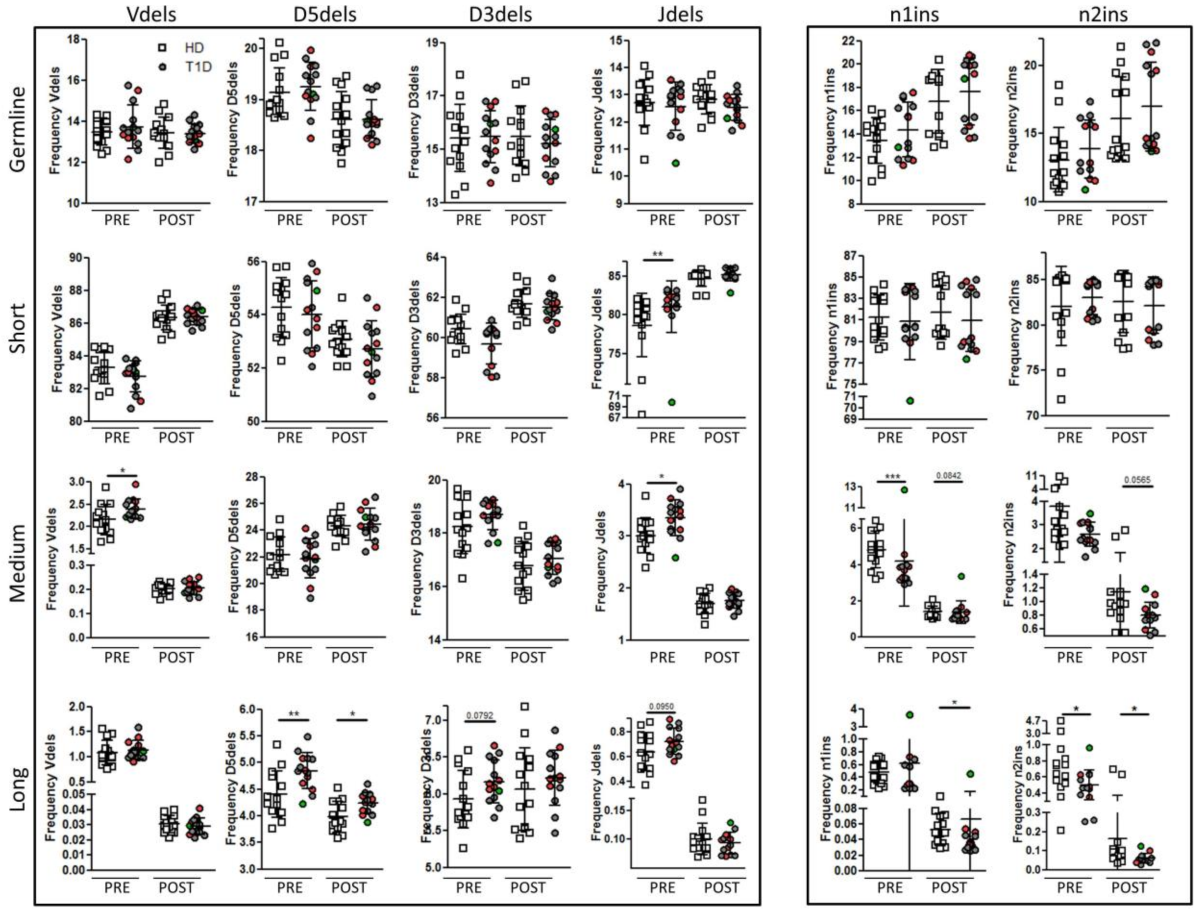

Supplementary Figure 12. Type 1 diabetes predisposing haplotypes do not explain the altered indel patterns. Data from Figure 5 plotted so that the values for germline, short, medium and long indels at each of the 6 rearrangement sites for each of the subjects can be compared. Shown in red are the *DRB1*03/DRB1*0401/DQB1*02/DQB1*03* type 1 diabetes patients to demonstrate that they are not outliers or unrepresentative of the study subjects. Green represents T1D #2, an outlier at the insertion sites (see Figure 5e). Unpaired t-test or Mann-Whitney U test. *: $p<0.05$. **: $p<0.01$. Lines represent the mean ± SD.

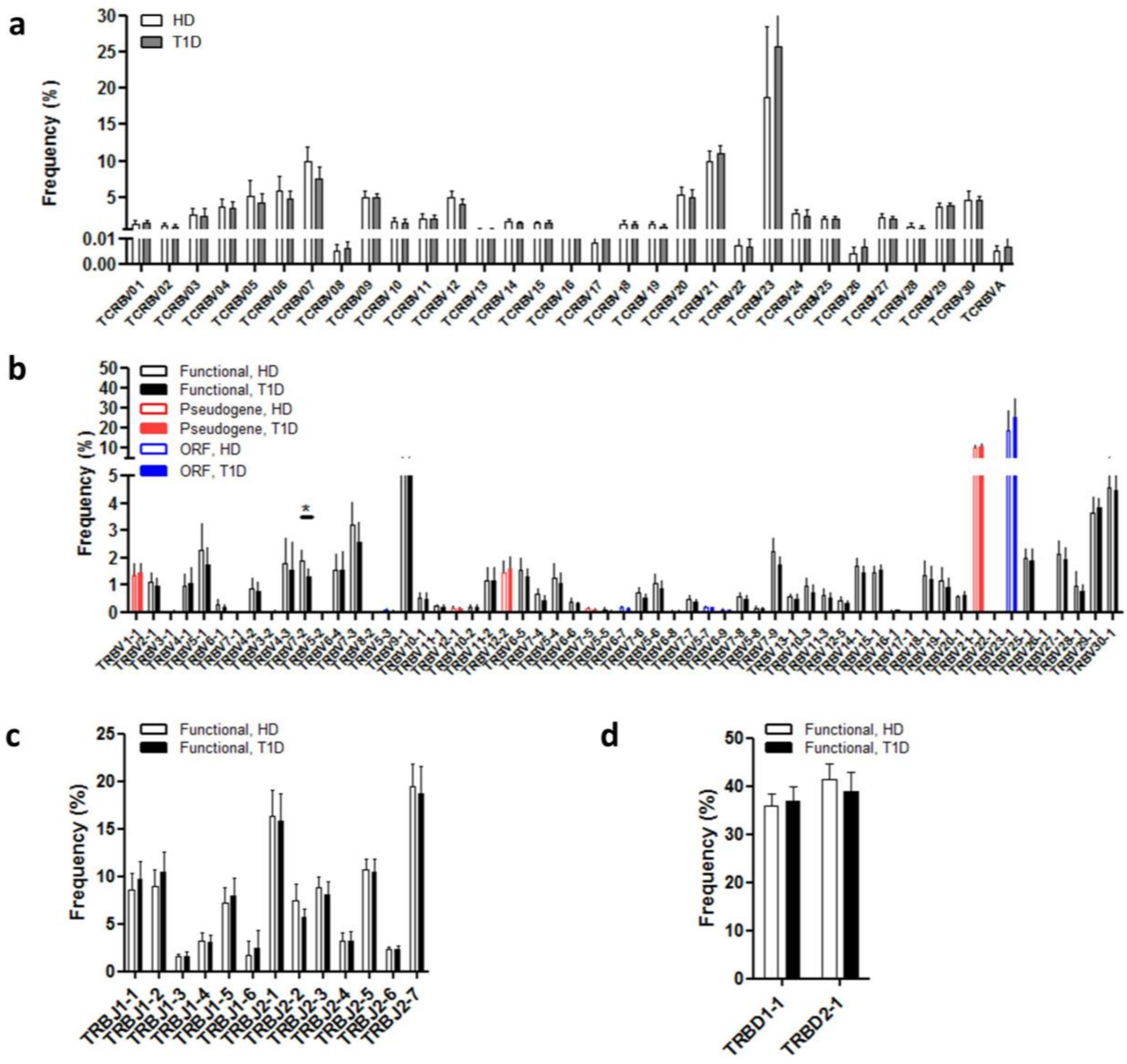

Supplementary Figure 13. *VDJ* gene usage in pre-selection TCRB repertoires. Frequency of use of each *V* family (**a**), *V* gene (ordered as located in the chromosome) (**b**), *J* gene (**c**) and *D* gene (**d**) in pre-selection sequences. **a**: Grey bars: Type 1 diabetes patients. White bars: healthy donors. **b,c,d**: Filled bars: type 1 diabetes patients; empty bars: healthy donors; red bars represent pseudogenes, blue bars open reading frame genes, and black bars functional genes. Mann Whitney U test corrected for multiple comparisons (Benjamini-Hochberg, FDR level = 0.05). *: $p<0.05$. Error bars represent the SD.

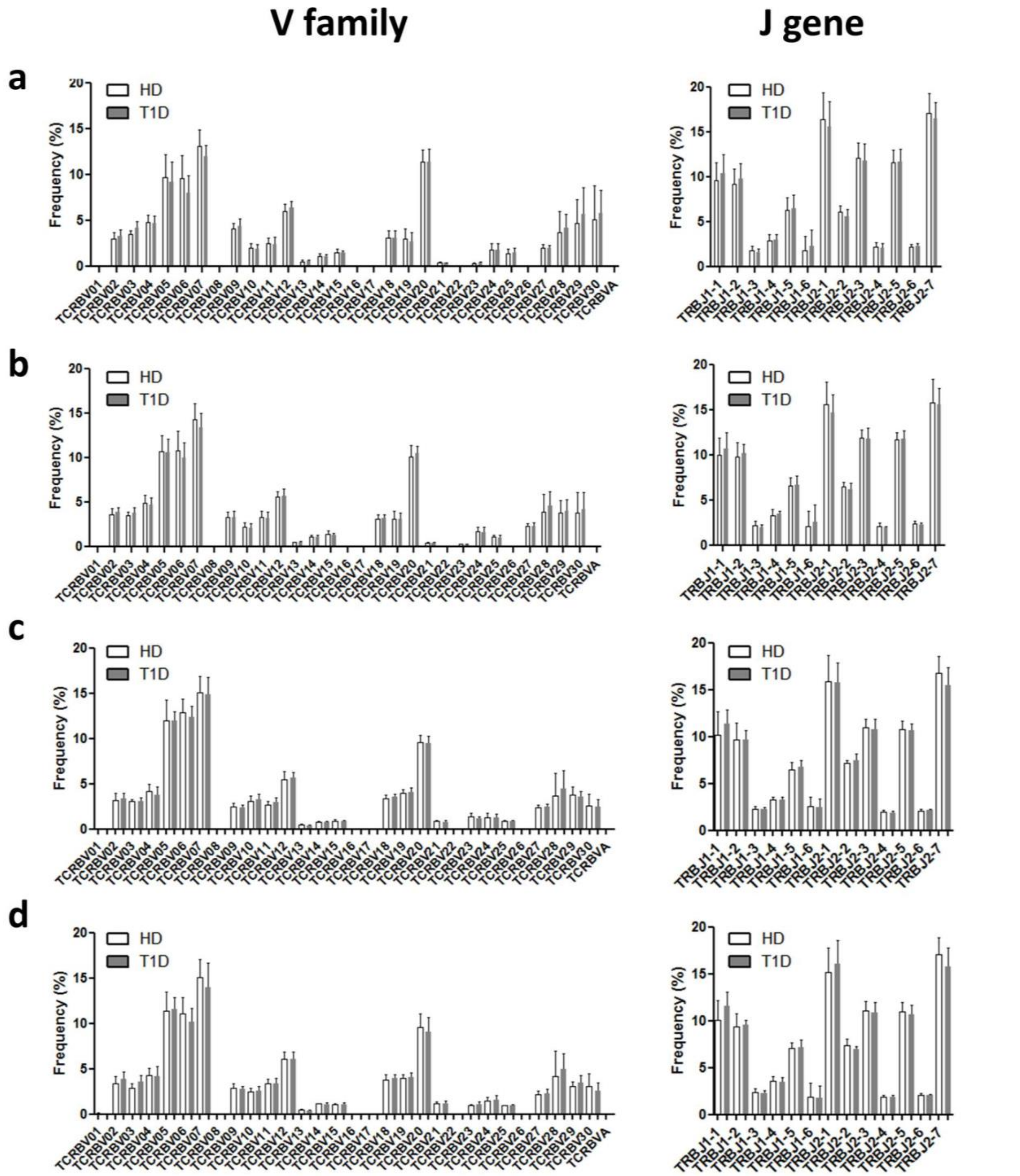

Supplementary Figure 14. *V* family and *J* gene usage in post-selection TCRB repertoires. Frequency of use of each *V* family (left column) and *J* gene (right column). There were no differences between type 1 diabetes patients and healthy donors. **a.** TN cells. **b.** CM cells. **c.** Treg cells. **d.** Tscm cells. Mann Whitney U test corrected for multiple comparisons (Benjamini-Hochberg, FDR level = 0.05). Error bars represent the SD.

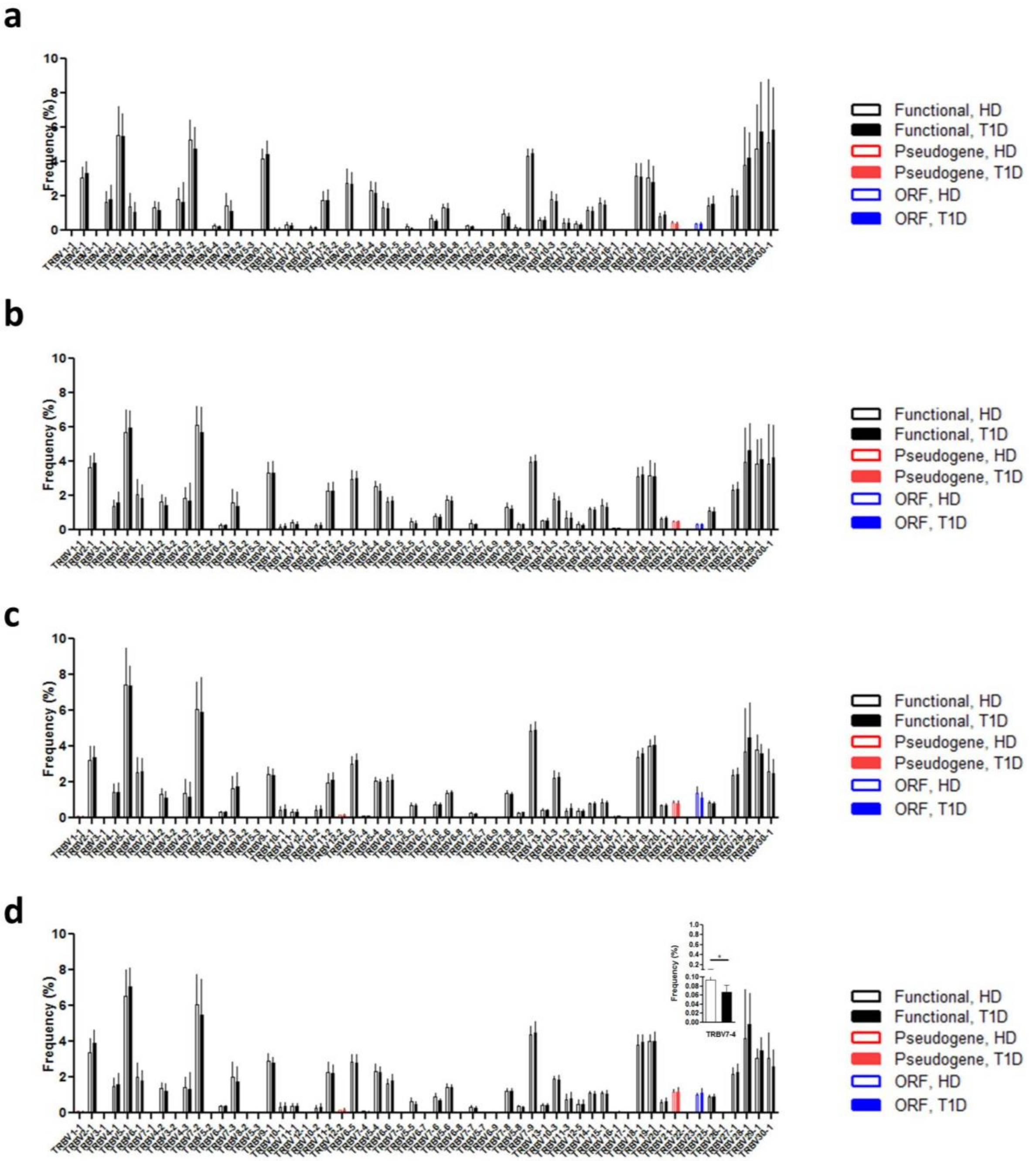

Supplementary Figure 15. *V* gene usage in post-selection TCRB repertoires. Frequency of use of each *V* gene (ordered as located in the chromosome; inset shows the statistically significant *V* genes). **a. True naïve (**TN) cells. **b.** Central memory (CM) cells. **c.** Regulatory T (Treg) cells. **d.** Stem-cell like memory T (Tscm) cells. Filled bars: type 1 diabetes patients; empty bars: healthy donors. Red bars represent pseudogenes, blue bars open reading frame genes, and black bars functional genes. Mann Whitney U test corrected for multiple comparisons (Benjamini-Hochberg, FDR level = 0.05). *: p<0.05. Error bars represent the SD.

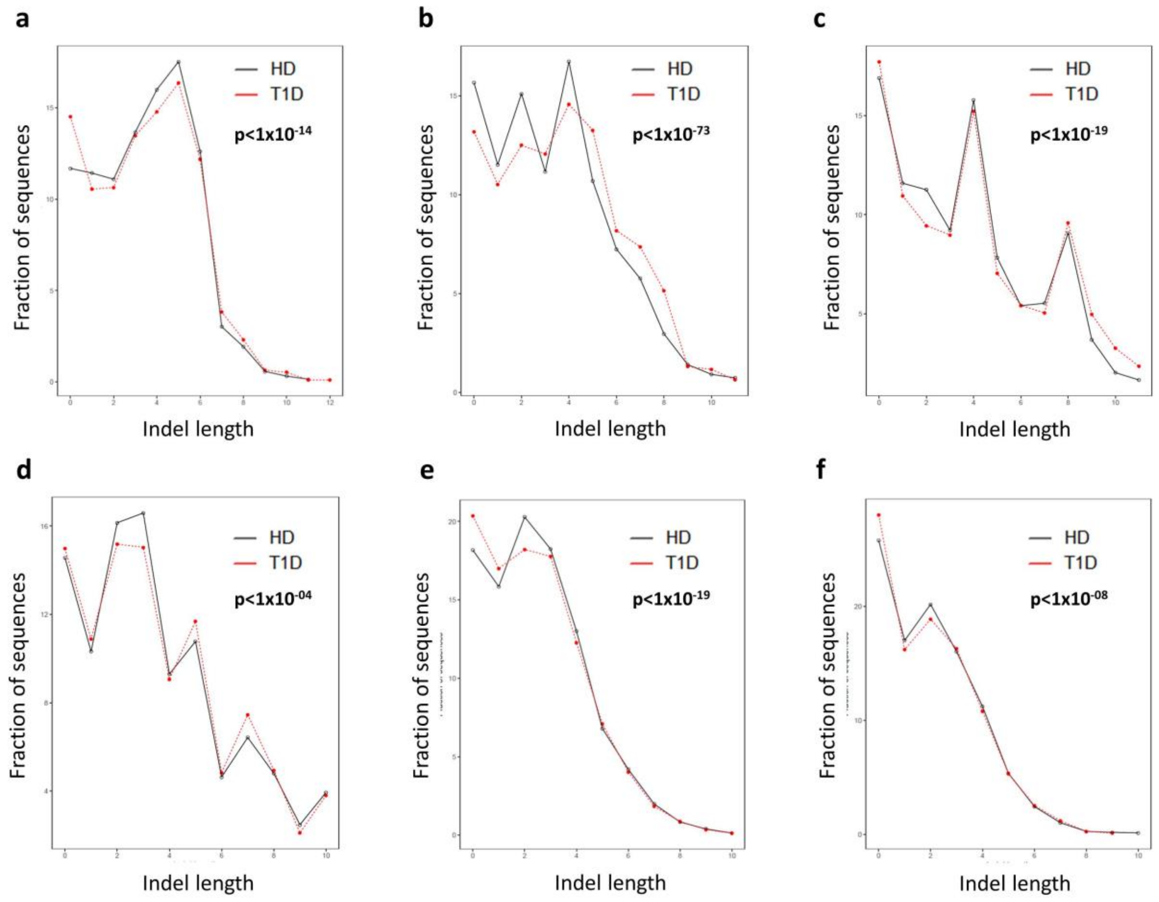

Supplementary Figure 16. Altered frequencies of deletions and insertions in T1D-exclusive clonotypes. Type 1 diabetes (T1D)-exclusive clonotypes show higher frequencies of long deletions and short insertions in all 6 rearrangement positions, when compared to healthy donor (HD)-exclusive clonotypes. **a.** Vdels. **b.** Jdels. **c.** D5dels. **d.** D3dels. **e.** n1ins. **f.** n2ins. One-sided bootstrap univariate Kolmogorov-Smirnov test. Black lines and dots: HD-enriched clonotypes.

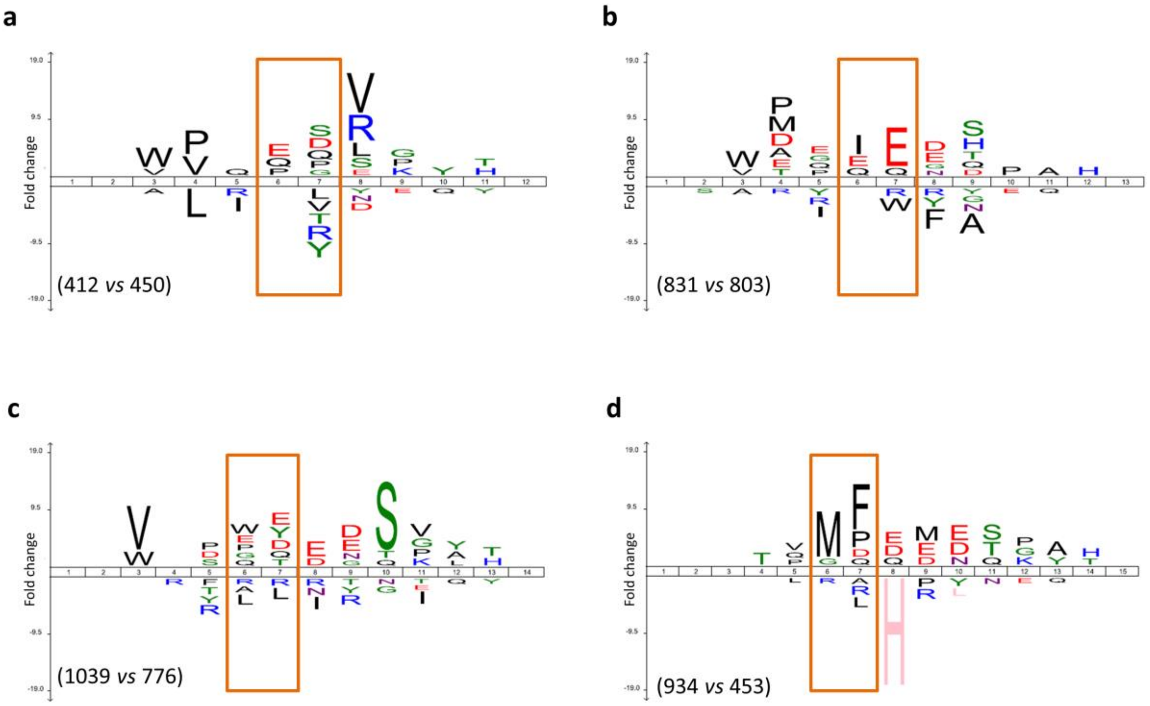

Supplementary Figure 17. T1D-enriched clonotypes show preferential amino acid usage. IceLogo plots for the more common lengths of the T1D-enriched and HD-enriched clonotypes (see Methods). **a.** 36 nucleotides (12 amino acids). **b.** 39 nucleotides (13 amino acids). **c.** 42 nucleotides (14 amino acids). **d.** 45 nucleotides (15 amino acids). Numbers in brackets show the number of HD-enriched and T1D-enriched clonotypes of that particular length, respectively. Orange squares highlight the positions P6 and P7 of the TCRB CDR3. The amino acids shown are those that are significantly different between T1D-enriched and HD-enriched clonotypes ($p<0.05$; see Methods). Over-represented amino acids in T1D-enriched: fold change > 0. Under-represented amino acids in T1D-enriched: fold change < 0).

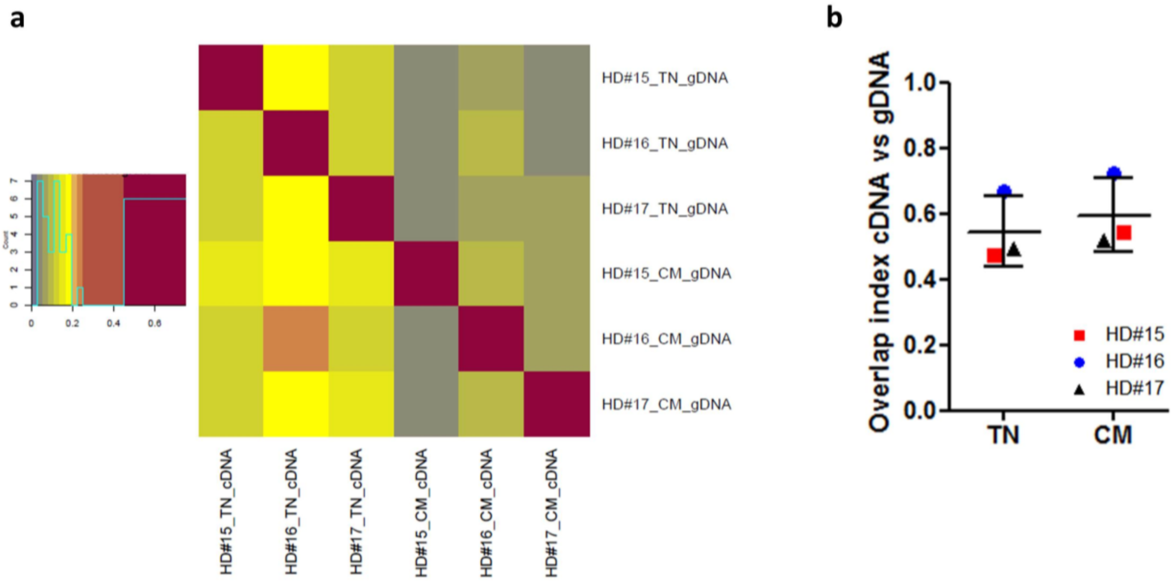

Supplementary Figure 18. Overlap indices between cDNA and gDNA show high repertoire sharing. True naïve (TN) and central memory (CM) cells from 3 healthy donors were sorted, RNA and genomic DNA (gDNA) isolated, cDNA synthesized, and cDNA and gDNA deep sequenced to compare overlap of the TCRB repertoires using these different nucleic acid materials from the same cells. **a.** Heatmap showing all the overlap indices of cDNA and gDNA samples. Overlap indices are high between cDNA and gDNA material from the same subject (main diagonal of the heatmap). **b.** Overlap indices between cDNA and gDNA shown in the main diagonal in **a.** Lines represent the mean ± SD.

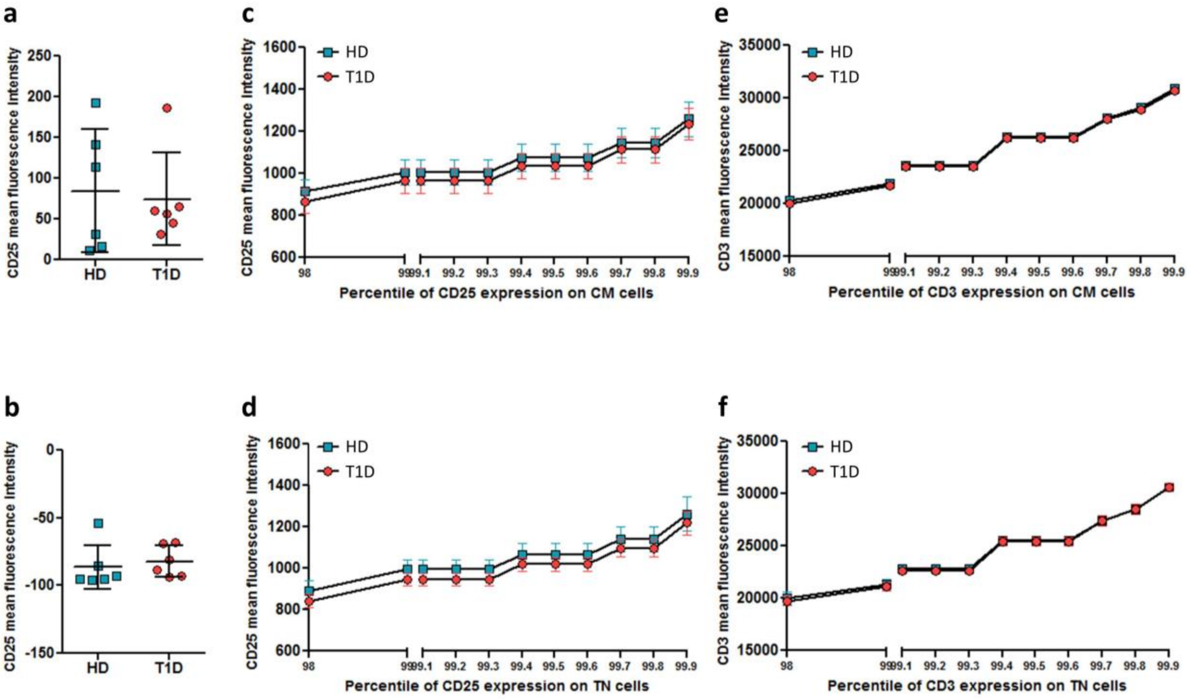

Supplementary Figure 19. Levels of activation and CD3 expression are similar in healthy donors and type 1 diabetes patients. a,b: the mean fluorescence intensity for the activation marker CD25 is similar between healthy donors (HD) and type 1 diabetes patients (T1D), both in central memory (CM) (**a**) and true naïve (TN) (**b**) cells. **c-f:** the mean fluorescence intensity for CD25 (**c,d**) or CD3 (**e,f**) was analysed in the top 2% of CD25 (**c,d**) or CD3 (**e,f**) expressing cells (98th, 99th and 99.1^{th}-99.9^{th} percentiles), showing no differences between healthy donors and type 1 diabetes patients. **a-f:** Student's t- or Mann-Whitney U test. Lines represent the mean ± SD. Data shown is for 6 healthy donors and 6 type 1 diabetes patients.

Subject	Age (years)	Gender	Months since diagnosis	*HLA-DRB1*	*HLA-DQB1*	Number of cells sorted ($x10^5$)				Number of productive unique reads ($x10^4$)			
						TN	CM	Treg	Tscm	TN	CM	Treg	Tscm
T1D #1	22	M	5.4	*03:01/06, 04:01*	*02, 03:02/05*	19.000	16.000	3.009	0.867	48.645	29.767	3.933	3.771
T1D #2	27	F	5.9	*04:01, 13:02/31*	*03:02/05, 06:04/05*	14.000	4.551	2.492	0.668	61.724	17.842	4.614	2.624
T1D #3	46	F	10	*04:04, 13:02/31*	*03:02/05, 06:04/05*	25.000	21.000	5.015	1.080	45.770	30.319	9.595	4.193
T1D #4	36	F	7.9	*03:01/06, 04:04*	*02, 03:02/05*	26.000	14.000	3.155	2.130	46.225	26.976	4.676	8.850
T1D #5	34	M	2.5	*03:01/06, 04:01*	*02, 03:02/05*	18.000	18.000	4.767	1.183	44.403	23.163	7.185	2.761
T1D #6	19	F	3.4	*04:01, 11*	*03:01/04, 03:02/05*	24.000	17.000	3.925	0.658	64.821	27.778	7.612	2.353
T1D #7	30	M	2	*03:01/06, 04:01*	*02, 03:02/05*	19.000	14.000	3.459	1.629	48.254	26.309	8.919	8.049
T1D #8	32	M	7.9	*04:05, 13:01/05*	*03:02/05, 06:03/07*	15.000	6.000	2.105	0.850	40.756	11.429	2.576	4.203
T1D #9	21	M	14.5	*04:01, 13:02/31*	*03:02/05, 06:04/05*	25.078	14.175	N/A	N/A	34.557	37.708	N/A	N/A
T1D #10	39	M	7.83	*03:01/06, 04:01*	*02 , 03:02/05*	20.000	17.787	N/A	N/A	33.136	51.505	N/A	N/A
T1D #11	35	M	5.1	*04:04, 13:02/31*	*03:02/05, 06:04/05*	27.613	25.845	N/A	N/A	23.554	49.347	N/A	N/A
T1D #12	32	M	3.25	*04:01, 04:04*	*03:02/05, x*	23.106	22.869	N/A	N/A	90.697	21.171	N/A	N/A
T1D #13	22	M	8.5	*03:01/06, 04:01*	*02 , 03:02/05*	23.384	13.215	N/A	N/A	90.653	21.166	N/A	N/A
T1D #14	29	M	6.9	*0404, 08*	*03:02/05 , 04*	24.697	8.862	N/A	N/A	98.644	25.321	N/A	N/A
HD #1	47	F	N/A	*04:01, 15*	*03:02/05, 06:02/11*	11.000	12.000	4.086	0.526	44.091	25.024	4.299	2.616

HD #2	28	F	N/A	*04:01, 14:01/04*	*03:02/05, 06:01*	23.000	14.000	4.507	2.590	61.016	26.624	10.308	14.272
HD #3	32	F	N/A	*04:04, 07*	*02, 03:02/05*	15.000	8.381	4.319	1.427	55.594	23.012	6.473	6.962
HD #4	26	M	N/A	*01, 04:01*	*03:02/05, 05:01/07*	15.000	15.000	6.403	0.753	62.086	25.756	25.339	3.234
HD #5	26	F	N/A	*03:01/06, 15*	*02, 06:02/11*	10.000	3.636	2.213	0.883	38.932	6.772	1.594	2.626
HD #6	26	F	N/A	*04:07, 07*	*02, 03:01/04*	29.000	23.000	4.327	3.702	64.942	31.917	7.423	19.103
HD #7	30	M	N/A	*01, 03:01/06*	*02, 05:01/07*	14.000	14.000	4.857	4.811	51.991	29.293	4.810	15.247
HD #8	29	M	N/A	*04:01, 13:02/31*	*03:02/05, 06:04/05*	14.000	8.484	2.101	1.125	25.257	23.333	1.011	2.216
HD #9	26	F	N/A	*03:01/06, 07*	*02 , x*	20.000	17.927	N/A	N/A	40.968	68.290	N/A	N/A
HD #10	20	F	N/A	*04:01, 13:03/04*	*03:01/04 , x*	20.000	19.506	N/A	N/A	48.818	127.618	N/A	N/A
HD #11	29	M	N/A	*11 , x*	*03:01/04 , x*	17.314	16.000	N/A	N/A	25.011	87.890	N/A	N/A
HD #12	34	M	N/A	*11, 1301/15/16*	*03:01/04, 06:03/07*	29.171	23.223	N/A	N/A	42.159	33.768	N/A	N/A
HD #13	28	M	N/A	*01:03, 03:01/06*	*02 , 05:01/07*	28.287	18.950	N/A	N/A	38.634	68.304	N/A	N/A
HD #14	31	M	N/A	*11 , 15*	*03:01/04, 05:02/04*	20.000	17.470	N/A	N/A	44.415	63.705	N/A	N/A

Supplementary Table 1. Characteristics of study subjects and cell analysis details

1st PCR	
VAR-B1	GAAACAAGAATAGAAGGAGATATTGTATGTWYTGGTAHMRWCAG
VAR-B2	GAAACAAGAATAGAAGGAGATATTGTAGTWYTGGTATCRACAAG
VAR-B3	GAAACAAGAATAGAAGGAGATATTGTASTYYWCTGGTACMDRSAG
VAR-B4	GAAACAAGAATAGAAGGAGATATTGTAATTGGTATCGACGTGTTATG
VAR-B5	GAAACAAGAATAGAAGGAGATATTGTATTTWTTGGTAYCRACAG
VAR-B6	GAAACAAGAATAGAAGGAGATATTGTAGTTTACTGGTATCRKMAG
VAR-B7	GAAACAAGAATAGAAGGAGATATTGTATTGGTATCAGCAGAATCAG
VAR-B8	GAAACAAGAATAGAAGGAGATATTGTATGTCCTGGTATCGACAAG
VAR-B9	GAAACAAGAATAGAAGGAGATATTGTATRTMCTGGTAYMRACARG
VAR-B10	GAAACAAGAATAGAAGGAGATATTGTAGTATTGGTACAAGCARARWGC
VAR-B11	GAAACAAGAATAGAAGGAGATATTGTATGTTCTGGTACYGTCAGC
VAR-B12	GAAACAAGAATAGAAGGAGATATTGTACTATTGGTACAGACAAATC
C-region 3' B	GTCGGGGTAGAAGCCTGT
**2nd PCR**	
C-region 3’ B-1	CTTTTGGGTGTGGGAGATC
HTSP_oligo	GAAACAAGAATAGAAGGAGATATTGTA

Supplementary Table 2. Oligonucleotide sequences of primers used for *TCRB* single-cell PCR.
